# Veno-venous ECMO: a synopsis of nine key potential challenges, considerations, and controversies

**DOI:** 10.1186/1471-2253-14-65

**Published:** 2014-08-06

**Authors:** David B Tulman, Stanislaw P A Stawicki, Bryan A Whitson, Saarik C Gupta, Ravi S Tripathi, Michael S Firstenberg, Don Hayes, Xuzhong Xu, Thomas J Papadimos

**Affiliations:** 1 Department of Anesthesiology, Wexner Medical Center at The Ohio State, University, 410 W 10th Ave, Columbus 43210, OH, USA; 2 Department of Surgery, Division of Critical Care, Trauma, and Burn, Wexner Medical Center at The Ohio State University, 410 W 10th Ave, Columbus 43210, OH, USA; 3 Department of Surgery, Division of Cardiac Surgery, Wexner Medical Center at The Ohio State University, 410 W 10th Ave, Columbus 43210, OH, USA; 4 Northeast Ohio Medical University, 4209 SR 44, PO Box 95, Rootstown 44272, OH, USA; 5 Cardiothoracic Surgery, Summa Akron City Hospital, 75 Arch St, Akron 44304, OH, USA; 6 Pulmonary Medicine, Nationwide Children’s Hospital, 700 Children’s Drive, Columbus 43205, OH, USA; 7 Department of Anesthesiology, The First Affiliated Hospital of Wenzhou Medical College, 2 Fuxue Road, 32500 Zhejiang, China

**Keywords:** ECMO, Respiratory distress syndrome, Adult, Veno-venous, Cannulation

## Abstract

**Background:**

Following the 2009 H1N1 Influenza pandemic, extracorporeal membrane oxygenation (ECMO) emerged as a viable alternative in selected, severe cases of ARDS. Acute Respiratory Distress Syndrome (ARDS) is a major public health problem. Average medical costs for ARDS survivors on an annual basis are multiple times those dedicated to a healthy individual. Advances in medical and ventilatory management of severe lung injury and ARDS have improved outcomes in some patients, but these advances fail to consistently “rescue” a significant proportion of those affected.

**Discussion:**

Here we present a synopsis of the challenges, considerations, and potential controversies regarding veno-venous ECMO that will be of benefit to anesthesiologists, surgeons, and intensivists, especially those newly confronted with care of the ECMO patient. We outline a number of points related to ECMO, particularly regarding cannulation, pump/oxygenator design, anticoagulation, and intravascular fluid management of patients. We then address these challenges/considerations/controversies in the context of their potential future implications on clinical approaches to ECMO patients, focusing on the development and advancement of standardized ECMO clinical practices.

**Summary:**

Since the 2009 H1N1 pandemic ECMO has gained a wider acceptance. There are challenges that still must be overcome. Further investigations of the benefits and effects of ECMO need to be undertaken in order to facilitate the implementation of this technology on a larger scale.

## Background

Acute respiratory distress syndrome (ARDS) is the acute onset of hypoxemia, accompanied by diffuse bilateral pulmonary infiltrates and the absence of left heart failure [1]. There are over 140,000 cases of ARDS in the United States each year, with a mortality rate of 22-41% [2,3]. As many as 20% of patients with ARDS succumb to refractory hypoxemia, and the quality of life among the survivors can be significantly affected for a number of years following the initial episode [4,5]. Moreover, the average medical costs associated with long-term care for ARDS survivors are approximately four times greater than those expended for a person in good health [6].

There has been some progress in the management of severe lung injury and ARDS in the past two decades, including important clinical trials such as the ARDSNET trial, investigating ventilator management techniques associated with improved outcomes in ARDS patients [7]. Among key findings of the most prominent clinical investigations is the association between high ventilator tidal volumes and worsening lung injury [7-11]. On the other hand, the use of lower tidal volumes may improve overall outcomes and mortality [7-11].

After the failure of conventional mechanical ventilation, airway pressure release ventilation (APRV) and high frequency oscillatory ventilation (HFOV) can sometimes be used as “rescue” therapies [12]. However, the number of remaining life-saving options becomes increasingly more limited as the severity of illness progresses [13]. Extracorporeal membrane oxygenation (ECMO) is emerging as one of these alternative, life-saving maneuvers, mainly thanks to its successful use in the 2009 H1N1 pandemic [14-17]. ECMO is a form of partial cardiopulmonary bypass (CPB) that can be employed in longer-term support of respiratory and/or cardiac function. This technology was derived and adapted from the CPB traditionally used during cardiac surgery. Early ECMO system designs used “bubble oxygenators” that were poorly suited for prolonged use because of their tendency to hemolyze blood. Membrane oxygenators now have replaced “bubble oxygenators” and have made the long-term use (weeks instead of days) of ECMO possible.

## Discussion

### Implementation of ECMO

The first report of successful ECMO support in an adult patient was published by Hill in 1972 [18]. ECMO can be implemented either as veno-arterial (VA) or veno-venous (VV) therapy. For complete cardiopulmonary support, VA ECMO is used while primary respiratory failure including severe oxygenation failure is usually treated with VV ECMO. Both VA and VV approaches require a pump that is capable of generating flow rates of 3–5 L · min^-1^ in order to ensure sufficient organ perfusion and oxygenation. This discussion will confine itself to VV ECMO used in refractory respiratory failure, including clinical indications of pneumonia (bacterial, fungal, viral, aspiration), status asthmaticus, traumatic pulmonary contusions, pulmonary embolism, as well as for secondary causes such as ARDS associated with overwhelming sepsis or systemic inflammatory conditions. Large bore cannulae drain venous blood that is pumped through an oxygenator where it is cleared of carbon dioxide, oxygenated, and then actively returned back to the patient’s circulation. While both ECMO and CPB use somewhat similar technologies in terms of vascular cannulation, tubing for blood flow, and even oxygenators and pumps, the fundamental difference is that CPB incorporates a reservoir for adjusting real-time, total blood volume. Because there is no such reservoir in ECMO, meticulous attention to de-airing is critical when establishing perfusion [19].

It is important to remember that hypoxemia, hypercarbia, and acidosis are very potent pulmonary vasoconstrictors and these patients often have evidence of acute right heart failure and pulmonary arterial hypertension. Addressing these issues with ECMO can often break the vicious and often fatal downward spiral of hypoxemia, hypercarbic (respiratory) acidosis, right heart failure, low cardiac output and the resulting metabolic acidosis from impaired oxygen availability at the cellular level. In fact, severe respiratory acidosis with hemodynamic instability requiring vasoactive support can be an indication for ECMO [20].

The most comprehensive guidelines on ECMO are published by the Extracorporeal Life Support Organization (ELSO) [21]. These guidelines address personnel, training, resources, use of ECMO, and quality assurance. In the subsequent sections, we will discuss the points that most challenge a new practitioner who is newly faced with care of the patient placed on VV ECMO and its clinical implementation, focusing on practical clinical applicability of this life-saving therapy.

### Challenge one: who is eligible for veno-venous ECMO?

Careful selection of ECMO candidates is critical because of potential complications and substantial costs. Indeed, the associated high mortality may lead to a perception of ECMO being an inefficient use of scarce resources [3,18]. According to the ELSO guidelines the use of ECMO should be considered when the ratio of PaO_2_ to FiO_2_ is <150, and ECMO is indicated when the ratio is <80. PaCO_2_> 80 mm Hg or end-inspiratory plateau pressure >30 cm H_2_O is also considered an indication for ECMO in patients with ARDS [21].

According to Brodie et al., more specific indications for VV ECMO in the case of ARDS include severe hypoxemia (as indicated in the ELSO guidelines) despite the addition of high positive end expiratory pressure (PEEP) at the 15–20 cm H_2_O levels for six hours in those who have reversible respiratory failure; uncompensated hypercapnea with a pH < 7.15 in the face of best practice ventilator mode manipulation; and extremely high end-inspiratory plateau pressures (40 cm H_2_O) [3]. Relative contraindications include PEEP levels > 30 cm H_2_0 for >7 days, FiO_2_> 80% for >7 days, limited vascular access, and terminal cancer or severe brain injury that cannot be reversed. Of course, those patients who cannot receive anticoagulation must be excluded. Further suggestions and guidelines on using ECMO to rescue patients with severe ARDS can be found in the ongoing EOLIA (Extracorporeal Membrane Oxygenation for Severe Revere Respiratory Distress Syndrome) study, conducted by Combes et al. [22], the use of the Murray score in the CESAR Trial [19], and a study by Zapol et al. [20]. The value of these trials is that the clinical expertise available at an ECMO center is more than the ECMO itself. The expertise of the anesthesiologists, intensivists, and respiratory technologists allows for a broader depth of practice than what is often supported by smaller referral centers. In any event, the guidelines above may conflict with the specific guidelines from other countries and institutions. For instance, age > 65 years is an absolute contraindication in some countries, while an absolute weight as opposed to BMI may be used [23]. Who actually receives ECMO will vary by situation, institution and geography [21,24]. While many of the contraindications and decisions for candidacy are institutional specific, there are some controversies. For body mass index (BMI), there are physical limits to who can be cannulated and supported. If one has a BMI greater than 35 kg/m^2^, the perfusion circuit may not be able to generate enough flow without high line pressures. The elevated line pressure will lead to hemolysis over time. Unfortunately, obesity is an epidemic and the proportion of the population in developed countries above a BMI of 30 is increasing. An absolute BMI may be a relative contraindication or soft limit, from center to center.

If a patient has been on maximal support for >7 days, the extent to when they may be recoverable in a time period before adverse events occur comes into question. Prolonged ECMO runs lead to hemolysis, elevated LDH, elevated bilirubin and liver function tests, and even carbon monoxide (CO) poisoning (In ECMO, hemolysis is frequently a problem, especially secondary to disruption of the hemoglobin molecule by mechanical trauma from the ECMO cannulas. When hemoglobin is degraded, free iron, biliverdin and CO are produced by heme oxygenase enzymes). This can in turn lead to a coagulopathy and need for replacement of oxygenators/circuits. In addition, prolonged illness can lead to multi-system organ failure. Additionally, withholding ECMO support may be considered in a patient with repeated suicide attempts without previous psychological treatment and/or support. There are several indices that have been studied (e.g., SOFA, APACHE) which can prognosticate on the degree of illness and their association with survival. They may have value in ECMO decision management. Once a practitioner or team have decided to place a patient on ECMO, a palliative care team should be consulted to help the patient and/or their family determine the goals of care.

### Challenge two: choosing the cannula type

**Figure 1:**
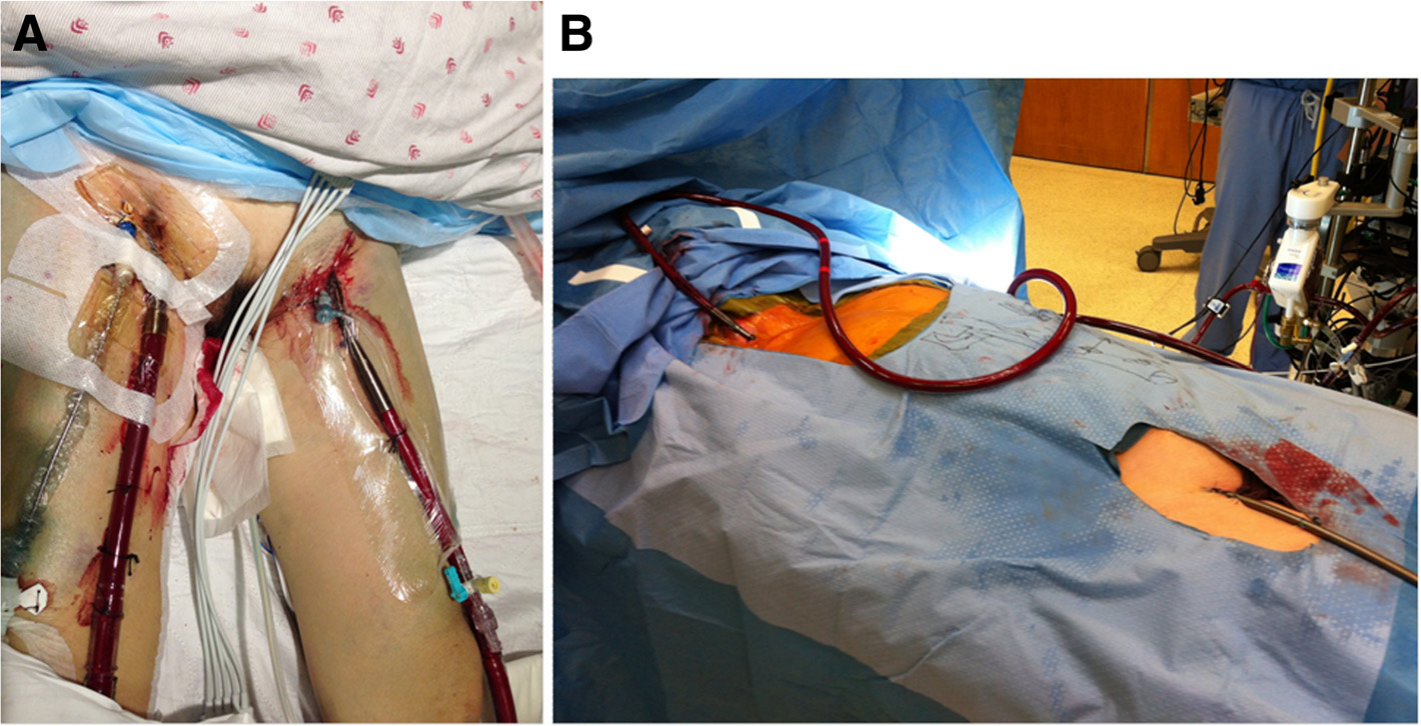
**Examples of VV femoral cannulization. (A)** femoral-femoral connection; **(B)** femoral jugular connection.

VV ECMO requires only venous access. Usually the access is accomplished using any combinations of femoral vein(s) (Figure 1), internal jugular vein(s) or subclavian vein(s). Ultrasound guidance in conjunction with the Seldinger technique may be helpful. VV ECMO can be administered with one cannula or two [25,26].

Typically, in situations involving ARDS, two catheters are placed via the femoral approach; however, this practice may vary from center to center. A drainage or outflow catheter is placed above the confluence of the iliac veins and the inferior vena cava and must be confirmed radiographically. The umbilicus may be used as an anatomical landmark externally. The inflow cannula is placed into the right atrium with the nipple as the external landmark. This allows blood drained from the abdomen and lower extremities to be oxygenated and returned to the right heart circulation. The final result is an optimization of systemic perfusion (normal cardiac function is required for this supposition).

The development of a bicaval dual lumen (BCDL) catheter (Avalon Laboratories, Rancho Dominguez, CA) has allowed for both inflow and outflow ports at a single site. However, the use of this technology can be challenging due to the complexity of catheter placement. Precise placement of the BCDL catheter is required to ensure correct direction of the reinfusion jet towards the tricuspid valve. BCDL catheter placement is often done with fluoroscopy and/or transesophageal echocardiography (TEE) [27,28].

**Figure 2:**
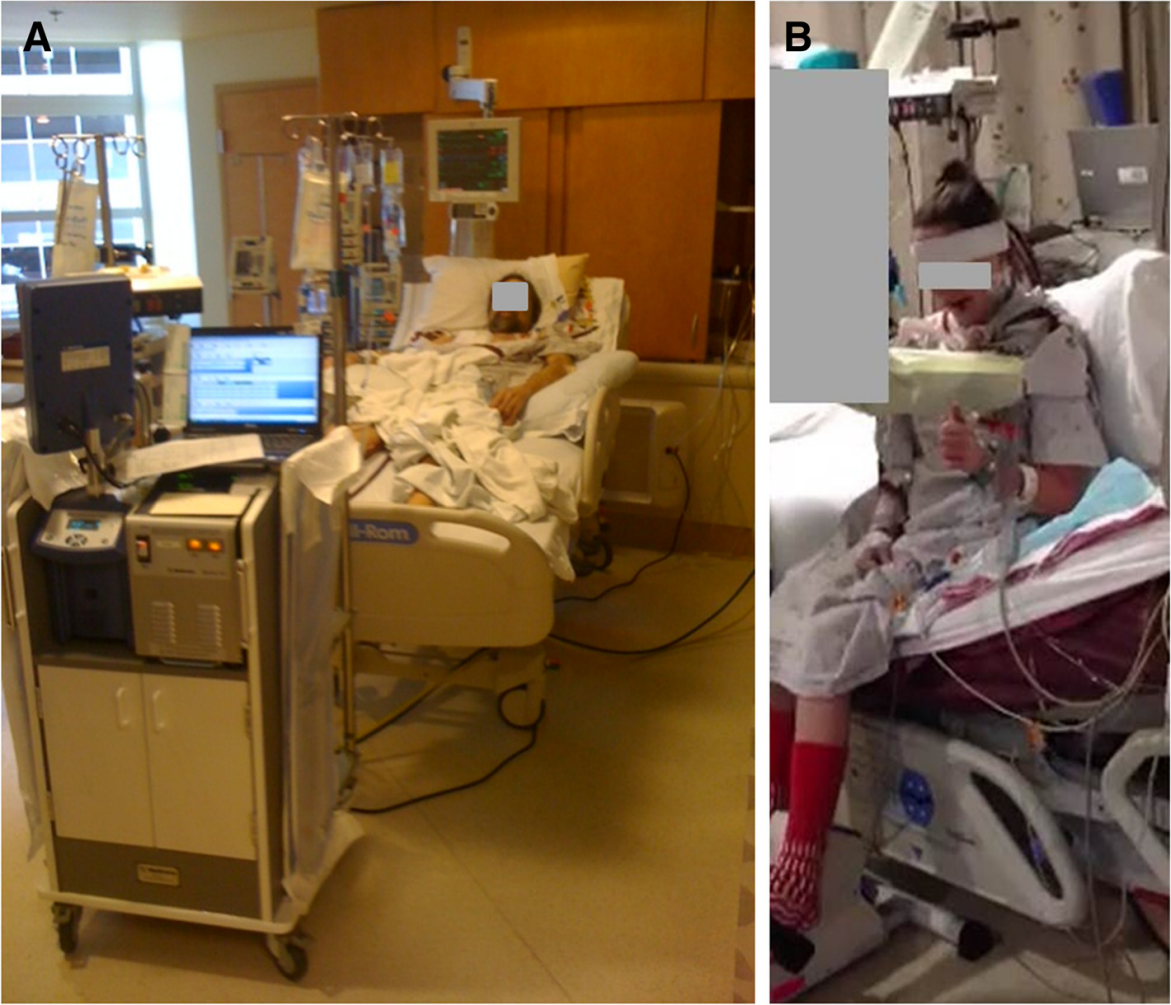
**Example of ECMO ambulation: (A) extubated**, **bed**-**ridden patient on ECMO; (B) patient on ECMO and able to sit up in bed and ambulate.**

There are both advantages and disadvantages to the BCDL catheter [28]. Advantages include (a) single insertion site, which decreases risk of bleeding and infection, (b) ability to ambulate patients (Figure 2), (c) posterior lateral position that is optimal for thoracic surgery, (d) permits prone positioning more easily, and (e) facilitates aeromedical transport. In contrast, the disadvantages include (a) limited range of catheter sizes with limited flows if inappropriate size inserted, (b) radiographic and/or echocardiographic guidance needed for insertion, (c) instability of catheter after placement, (d) insertion of a large, double lumen cannula into the internal jugular artery may result in cerebral venous congestion; an additional cephalad drainage line may be worth considering, and (e) the need for expertise in ECMO management for optimal outcomes. We use the BCDL frequently because we try to keep our ECMO patients ambulating and using a stationary bicycle type device whenever possible, recovery permitting.

### Challenge three: oxygenators and pump design

The main components of the ECMO circuit are the oxygenator and the pump. Draining from the outflow cannula, blood enters the pump and is driven through the oxygenator. The oxygenated blood is returned to the patient via the inflow cannula [29]. The circuit has three primary modifiable settings that are (a) the pump speed, which determines the flow of blood through the circuit; (b) the gas “sweep” that determines the level of gas exchange with the blood and hence assists in carbon dioxide removal; and (c) oxygen setting, much like the ventilator, is the amount of oxygen supplementation to the blood passing through the circuit. Control and monitoring of the system are done with a computer, which governs flows and driveline pressures with gas regulators to adjust oxygen support and carbon dioxide removal. External cooling and heating systems can be incorporated for clinical purposes, such as induced hypothermia.

Previous oxygenators were made of silicon membranes, whereas newer oxygenators have multiple hollow fibers of diameters less than 0.5 mm that are coated with polymethylpentene which allow for diffusion of gas, but not liquid. The newer oxygenators are more efficient (with less volume needed), more effective in gas exchange, cause less platelet and plasma protein loss, and even have thrombo-resistant coatings. However it is still important that pre and post membrane pressures are monitored in order to identify thrombus formation and the potential for membrane failure. With the use of polymethylpentene hollow fiber membranes, once the difference pressure across the membrane begins to increase it may progress more rapidly than previously seen in the older silicone plastic membranes, resulting in the need for emergent membrane replacement.

The most popular pump technology in use is the centrifugal pump. Traditionally, pumps had propellers driven by external motors. However, the heat generated from friction contributed to complications such as hemolysis, and in rare cases, melting the plastic of the pump itself. This problem has been resolved in newer pumps by the use of an impeller and suspending components of the pump magnetically, which minimizes direct contact and friction [30], thereby also allowing the use of small bearings. Be aware that centrifugal devices are pre-load and after- load sensitive pumps, i.e., the flow rate varies with the drainage volume and the pressure head being encountered. The traditional roller pumps have a constant flow and can fail or perform poorly secondary to high line pressure and inadequate drainage.

### Challenge four: transporting ECMO patients

**Figure 3:**
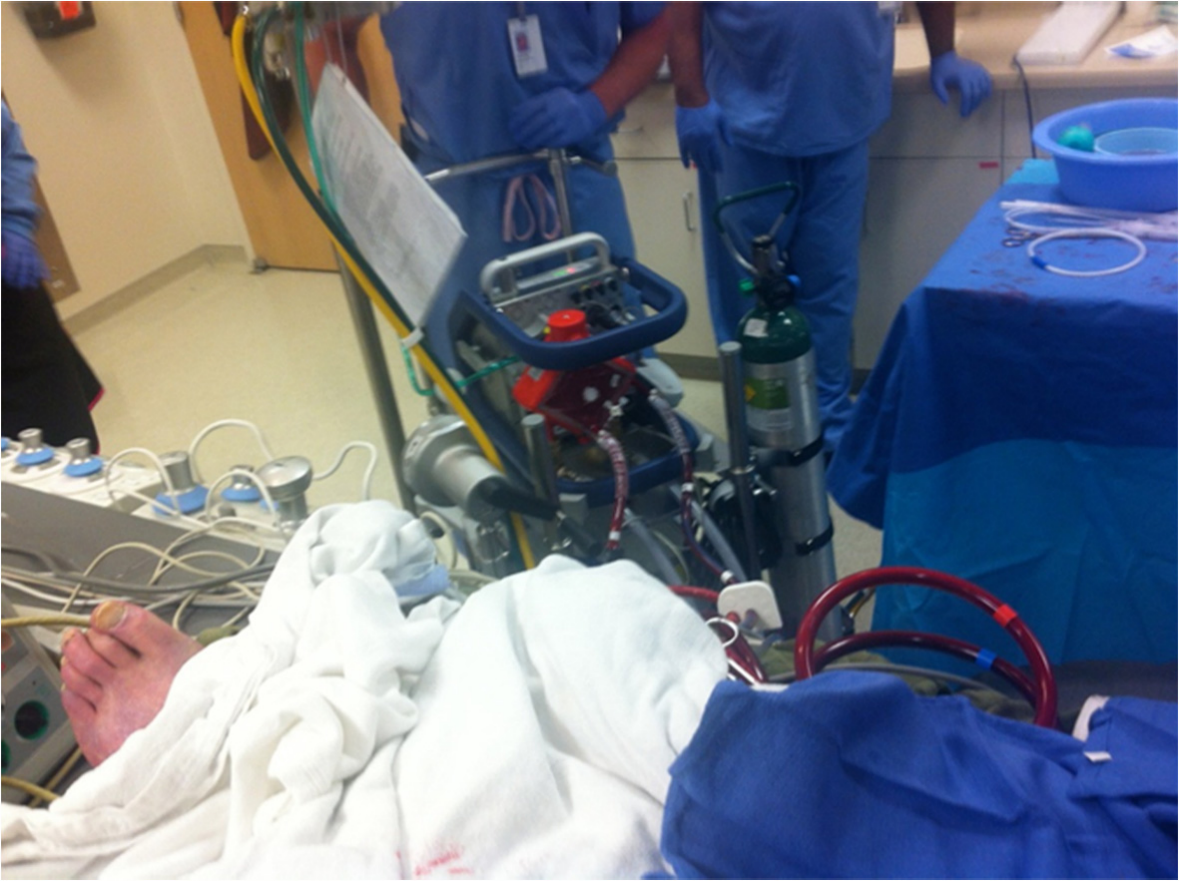
Maquet Cardiohelp portable device.

Improved engineering designs have significantly reduced the footprint of ECMO units and circuits, making travel much easier and more convenient [31]. Transport of ARDS patients, especially in military conflict can be challenging, let alone moving a small child or infant any distance. In centers with limited ECMO expertise, inter-hospital transfer may be a necessity. Optimally, patients who may require ECMO should be identified early and transported to a referral center (or center of excellence) that provides ECMO expertise. The practitioner must always be aware that the new transport technologies present a significant cost that may or may not necessarily be of benefit to the patient [32]. For example, in the Maquet Cardiohelp (22 pounds) portable device (Figure 3), the oxygenator and pump are one unit and can “clot” together, which may be problematic. Other modular ECMO may be used, such as the Lifebridge devices (the B2T model weighs 36 pounds), but always recognize the logistics of transport are exceedingly cumbersome [33]. Other devices include the configuration of the older Medtronic system, the Maquet Rotoflow, and the Thoratec CentriMag system.

The United States Air Force has assembled Critical Care Air Transport Teams (CCATT) that ferry critically ill and injured patients with lung injuries from combat/disaster areas. Combat related thoracic injures and ARDS led to a refinement of the CCATT concept which evolved into The Landstuhl Acute Lung Resue Team (ALerT) that uses pumpless extracorporeal lung assistance when necessary to transport patients [34,35]. This type of transport has also been duplicated successfully between children’s hospitals recently in Columbus, Ohio [36]. It is evident that protocol driven rescues are taking place, and while the protocols may vary from organization to organization, the miniaturization of devices has changed the face of transport dramatically.

### Challenge five: transfusion and intravascular fluid management

The absence of the reservoir for real-time adjustments of blood volume in VV ECMO permits lower levels of anticoagulation that minimize clotting of the circuit. However, the lack of this reservoir creates a potential disadvantage in that more active management is required in order to optimize the patient’s intravascular volume status. Consequently, ECMO patients require infusion of additional fluids via central or peripheral intravenous access, or via the ECMO circuit itself when additional volume is needed. Volume management in VV ECMO is assisted by the fact that the circuit has a heating ability (and also a cooling ability to allow for neuroprotection in the event of a cardiopulmonary arrest or a severe febrile state).

In VV ECMO, mechanical ventilation is minimized and hematocrit is kept at around 40%; SpO2 of 80% and PaO2 of 40 mm Hg are acceptable (the basis for this statement can be reviewed in the ELSO guidelines and by a review of the equations involved with the oxygen content of arterial and venous blood). However, the benefit gained from maintaining high hematocrit has to be carefully considered in the context of known harmful effects of transfusion [37,38]. Blood is associated with increased mortality [39], transmission of viruses [40], and greater risk of infections in general [41]. Brodie et al. recommend using the same transfusion thresholds as those used in the care of patients with ARDS who are not being treated with ECMO [3].

There are several indicators of volume depletion in a VV ECMO patient. When intravascular volume is depleted or compromised the ECMO cannulas swing back and forth (chattering of lines). The traditional response has been to give fluid, or in the case of a hematocrit of less than 40%, to transfuse packed red blood cells. Additionally, in regard to volume status, monitoring of the relative changes in a central venous pressure line may be of help. Additionally, for centrifugal pumps, the fluctuating of the flow rate markedly over a rather short period of time (second to minutes) may be foretelling. Also, simply the documentation of a decrease flow may assist in decision-making. This appears to occur because the cava/atrium is being sucked down on the cannula and the speed needs to be cycled (decreased then ramped up again) to correct this situation (as you would do if your vacuum cleaner sucked up a rug); such patterns indicate hypovolemia or excessive pump speed or cannula malposition. Be aware that an increase of 10% body weight secondary to fluid accumulation leads to increased mortality [42]. This approach leads to an accumulation of fluid that may be removed with diuresis/pharmacologically or by the use of renal replacement therapy. Our experience is to maintain a slightly negative fluid balance as tolerated by the patient.

Transiently decreasing the output of the pump is preferable over administration of intravenous fluid if extracorporeal blood flow is compromised by depletion of intravascular volume [3]. This approach may require intermittent increases of the ventilator support (i.e., FiO_2_) to maintain adequate oxygenation in the face of temporarily lower blood flows within the ECMO circuit. This maneuver can be reversed once the intravascular volume is restored and/or optimized.

### Challenge six: anticoagulation

Anticoagulation is necessary during active ECMO therapy because of the high thrombogenicity associated with blood coming into direct contact with the extracorporeal tubing. Bleeding and thromboembolic events may be the most frequent causes of death [43]. Most ECMO tubing circuits are heparin-bonded which can minimize the coagulation risk or provide some antithrombotic protection without systemic anticoagulation for short periods of time. Heparin bonding typically wanes after 1–2 days, which might be enough time to control surgical bleeding or reduce the risk of initial bleeding complications in high-risk patients. Typically heparin is used with conventional nomograms of dosing. Usually an aPTT is targeted to the range of 60–80 seconds, but this varies from institution to institution. One major drawback is that heparin leads to heparin-induced thrombocytopenia (HIT) syndrome in 10-15% of ECMO patients [44,45].

Another anticoagulation option is bivalirudin, which was first used successfully in ECMO in a patient with acute HIT [46]. Bivalirudin may result in less clotting of the circuit and fewer bleeding complications. In 21 ECMO patients receiving bivalirudin, Ranucci et al. demonstrated the efficacy of bivalirudin compared to heparin with regard to coagulation profile, less bleeding associated with bivalirudin, and the safety of bivalirudin as the sole coagulant for postcardiotomy ECMO [47]. Patients who received bivalirudin received fewer allogenic transfusions and experienced reduced costs compared to heparin. Due to the small numbers and retrospective nature of the study, its impact is limited and further investigation of bivalirudin use in ECMO patients is warranted. Practitioners must be aware that direct thrombin inhibitors have a high cost and do not have an antidote (reversal agent) [47].

One future option may be to avoid the use of anticoagulant. A newly developed circuit allowed 151 days of ECMO in an animal model with a trivial amount of heparin [48]. The development of the T-NCVC coating system, a complex of bonded heparin and aliphatic coupling reagents, may prevent heparin from leaching into the ECMO system. When the material comes in contact with blood, heparin is retained on the surface of the material. The activated clotting time (ACT) and aPTT were kept in the physiologic range and plasma heparin was not detected until the 21^st^ week. Platelet levels decreased for the first 4 weeks, but then remained constant. No elevation of plasma free hemoglobin occurred and hemoglobin levels were maintained through the 20^th^ week. The pump and oxygenator were essentially free of thrombus. The study had to be terminated because hemolysis occurred after 151 days on ECMO and there were problems with the centrifugal pump.

### Challenge seven: the airway

The practitioner should remember that there is little doubt that the patient with severe ARDS requiring ECMO should remain intubated with tracheostomy as a viable option for patient comfort and ease of care of the airway. However, the necessity of keeping VV ECMO patients intubated is being investigated, especially since the modern bicaval dual lumen catheter facilitates ambulation. In centers with the appropriate experience, patients waiting for lung transplantation are extubated and encouraged to ambulate and participate in active rehabilitation [49,50]. Participation in physical therapy in this manner prevents deconditioning and improves long-term outcome [51]. Ultimately, patients may not require endotracheal intubation to survive, although an established airway facilitates bronchoscopy and airway clearance. In fact, it has been recently demonstrated retrospectively that patients do not have to be intubated during bridging to lung transplantation and can be managed successfully in this manner [52,53]. Lastly, it is important to remember that the duration of pre-ECMO intubation has been associated with lower likelihood of decannulation [54].

### Challenge eight: neuromuscular blocking agents

Neuromuscular blocking agents (NMBAs) have been controversial in regards to their efficacy in treating ARDS. Due to lack of evidence on a large scale, no clear recommendations exist regarding the use of NMBAs in ARDS. However, some studies suggest that anesthesia and paralysis cause a ventilation/perfusion mismatch and impair gas exchange [55].

Recent developments assert that the hypoxemia in ARDS reaches its worst levels in the first 48 hours. The traditional view on NMBA use in the critical care setting is largely negative, with a number of potential complications associated with this therapeutic modality [56-58]. In a study of 56 patients with ARDS, improved oxygenation was seen in patients randomized to NMBAs in the first 48 hours while receiving volume assist control with a tidal volume of 6–8 ml/kg [59]. Another similar study reported that early NMBA use may contribute to modulation of the pro- inflammatory response [60]. Additionally, a third study of 340 patients where cis-atracurium was administered in the first 48 hours of development of ARDS found that the NMBAs improved the adjusted 90-day survival and increased time off of the ventilator without increasing muscle weakness [61]. Neto et al. performed a systematic review of the literature and meta-analysis of studies conducted between 1966 and 2012, and the three above-mentioned studies were the only acceptable, high-quality trials performed [62]. The authors concluded, based on these three studies, that that the use of NMBAs in the early stages of ARDS leads to an improved outcome. Nonetheless, further trials may need to be performed to solidify such a conclusion. The general sedation of the VV ECMO patient is beyond the scope of this synopsis, but sedation of the patient requires forethought and constant reevaluation. Never forget to sedate a patient in which a NMBA is used. As indicated, the above suppositions related to NMBAs were not directly related to ECMO, but difficulty in oxygenating an ECMO patient should at least lead to the consideration of pharmacologic paralysis.

### Challenge nine: the hospitals

Analyzing the differences across medical centers is a challenge in itself, especially since level of expertise can widely vary even within a single hospital. The CESAR Trial and corresponding analysis compared conventional practice and ECMO treatment in only one institution [63,64]. Ninety ARDS patients were randomized to receive “best practice” conventional treatment, and 90 were randomized to receive VV-ECMO. Three-quarters of the patients who were randomized to ECMO at a referral care center. Of the 90 transfers to the ECMO center, 17 did not need ECMO because the staff at the referral center was more effective in ventilator management. Of those that were stabilized through expert use of mechanical ventilation, 14 out of 17 survived. More patients were alive at 6 months than the control group (not of statistical significance), but more survived without disability. Referral for ECMO was also associated with .03 quality-adjusted years at 6 months.

This trial suggested that patients on ECMO do better at hospitals that are more experienced with ECMO. This conclusion is further supported by subsequent studies. Freeman et al. studied the impact of ECMO patient volume on mortality in the pediatric and neonatal population [65]. They retrospectively examined 7322 pediatric patients less than 18 years of age and demonstrated that low annual volume is associated with a higher mortality. Furthermore, they suggest that a minimum threshold volume be established to define a regional facility as a center of excellence (a volume of 22 ECMO patients was needed for excellence in care). Similar findings of the importance of ECMO patient volume were also reported by Karamlou et al. Karamlou and colleagues reviewed 3867 cases of ECMO that were identified in a pediatric congenital heart surgery program. They also suggested development of regional centers of excellence [66]. Michaels et al. demonstrated the same advantage in regard to adults [67]. Finally, Davis et al. concurred that the use of specialized centers with adequate patient volume led to good ECMO results in children in need of diaphragmatic hernia repair [68].

Improved pump design and membrane technology, along with simplified circuit design have prompted many hospitals that do not offer ECMO to place patients on bypass with the intention of transporting them to ECMO referral center (centers of regional excellence). The decision to provide ECMO, in effect, is taken out of the hands of ECMO experts, and this may result in patients who may have otherwise been excluded receiving ECMO support. On the other hand, patients may be placed on ECMO, who may have otherwise been managed by more conventional means by experts at centers of excellence. Positive outcomes are facilitated if the center of ECMO expertise assists in the training of peripheral hospitals in providing the initial training and support of such staff.

## Summary

Choosing who receives ECMO treatment is outlined in ELSO protocols, but there are many considerations that must be weighed as indicated in this synopsis. Essential considerations are the futility of treatment and the safety of anticoagulation. There are a variety of other issues that are not as widely agreed upon, and are left to the discretion and experience of the treatment team. Each patient must be evaluated in the context of specific co- morbidities and conditions present, ranging from uncorrected surgical/anatomical problems, morbid obesity, and any other relevant concerns. Another critical, overarching message that needs to be considered before placing a patient on ECMO support is the fact that one of the most significant mortality risks associated with this therapy is the extra pulmonary organ function at the time of initiation of ECMO therapy [69].

Using a single cannula with a branched inflow-outflow design BCDL catheter has many advantages over the more traditional two-cannula approach [70]. Currently, the technology and development state of the single-site ECMO support is the primary restriction that is limiting wider use. First and foremost, there are a limited number of ECMO-capable facilities that can manage ECMO via a bicaval dual lumen catheter [71,72]. Additionally, there are restrictions regarding the catheter itself, specifically the limited size availability that can lead to complications/limitations in flow. The complexity of placement with the need for radiographic and/or TEE guidance remains to be a hindrance, and the answer to this particular challenge may be wider adoption and implementation of high-fidelity ECMO simulation [73-75].

Pump design in ECMO technology has vastly improved [76], which has resulted in resolution of technical complications, such as hemolysis as a result of heat from the pump components [77,78].

However, centrifugal pumps are not flawless despite their advances. They are still preload and afterload sensitive. If there is a downstream occlusion of the pump circuit (afterload increase), the centrifugal pump will continue to spin but will not move blood volume forward. Likewise, if there is a reduction in available drainage/inflow volume or any occlusion of the inflow cannula, the pump will suck down on the tubing and generate significant negative pressures that will result in hemolysis and, more importantly, cause an acute drop in blood flow. However, these events can often be managed by reducing the pump flow/speed to decrease or eliminate the suction process and allow time to troubleshoot the cause [78].

Anticoagulant is an indispensable part of the ECMO process, requiring frequent monitoring and review [79]. The current standard is heparin, but bivalirudin is a promising alternative that can be easily implemented. Despite the fact that conclusive evidence on bivalirudin is still quite limited, the few studies conducted, albeit retrospectively with small sample sizes, suggest that bivalirudin is a superior alternative to heparin [46]. Caution still needs to be exercised when using bivalirudin, especially since a number of potential complications of this therapy have been reported [80,81]. The use of argatroban has also been described in the setting of HIT, as outlined by Mejak et al. [82]. Experimentally, an ECMO circuit using trivial amounts of anticoagulant has proven interesting. There may be limited need for anticoagulant by using a new type of coating, [83,84]. The equipment is still in testing phases but may hold great promise in the future [85].

Differences in ECMO expertise and preferences across hospitals continue to vary. Consequently, patient outcomes may not be fully reflective of the technology itself. In centers of excellence where the staff are considered experts in both mechanical ventilation and ECMO, patients have a likelihood of a positive outcome. In cases where chances of mortality are extremely high, ECMO is certainly an appealing option [63]. Unfortunately ECMO’s usage in “last-resort” type situations can often overvalue its lifesaving potential if the pretreatment mortality prediction is overestimated [69,86].

There is evidence that further investigations addressing paralysis with NMBAs in patients with ARDS treated with ECMO should be pursued [61,87,88]. Unfortunately, the effectiveness in the improvement of oxygenation or lung function was not clearly addressed in these reports, so a consensus on NMBAs remains controversial. The majority of the current research focuses on long term effects rather than the benefit of NMBAs during the first few days of ARDS. It is suggested that the worst hypoxemia occurs during this early period and thus a major reason why NMBA’s may play a pivotal role in recovery.

ECMO was successfully used in the 2009 H1N1 pandemic and subsequently gained a wider audience, demonstrating great potential in ameliorating patient outcomes. There are still a myriad of unique challenges that must be overcome and understood in the ongoing development process for advancing and standardizing ECMO practices. Many of the issues plaguing the early advance of ECMO have seen technological progression and resolution making it a much more viable, and efficient option. Further study needs to be undertaken in order to better realize the benefits and effects of ECMO as well as improve the implementation of the technology on a wider scale.

### Consent

Written consent to publish the featured images was obtained from each patient.

## Competing interests

The authors declare that they have no competing interests.

## Authors’ contributions

DBT, SPAS, BAW, SCG, RST, MSF, DH, XX, TJP All authors performed a literature review and participated in the drafting and editing of the manuscript content. All authors have reviewed and agree with the content of the final submitted manuscript.

## Pre-publication history

The pre-publication history for this paper can be accessed here:

http://www.biomedcentral.com/1471-2253/14/65/prepub
